# 3-Butyl-1-methyl-1*H*-imidazol-3-ium bis­(1,2-dicyano­ethene-1,2-dithiol­ato-κ^2^ *S*,*S*′)nickel(III)

**DOI:** 10.1107/S1600536811046824

**Published:** 2011-11-09

**Authors:** Shan-Shan Yu

**Affiliations:** a School of Biochemical and Environmental Engineering, Nanjing Xiaozhuang College, Nanjing, 210017, People’s Republic of China

## Abstract

In the title compound, (C_8_H_15_N_2_)[Ni(C_4_N_2_S_2_)_2_], the Ni^III^ atom is coordinated by four S atoms of two maleonitrile­dithiol­ate ligands and exhibits a distorted square-planar geometry. In the crystal, the cations and anions are connected alternately by weak inter­molecular C—H⋯N hydrogen bonds, forming a zigzag chain along [201].

## Related literature

For applications of bis­(1,2-dithiol­ene) complexes of transition metals, see: Nishijo *et al.* (2000 ▶); Ni *et al.* (2005 ▶). For related structures, see: Ni *et al.* (2004 ▶); Ren *et al.* (2004 ▶, 2008 ▶); Duan *et al.* (2010 ▶).
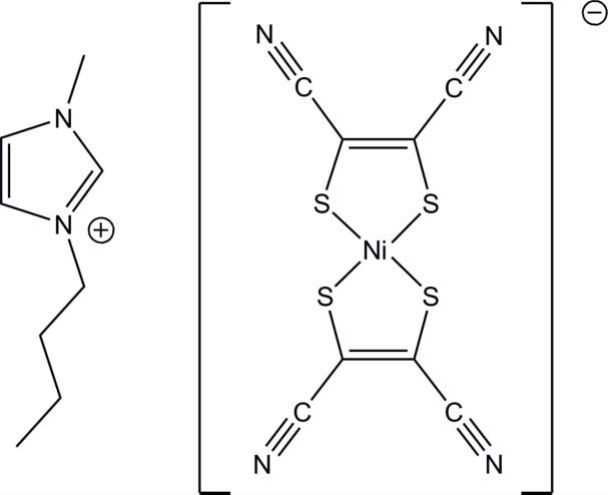


## Experimental

### 

#### Crystal data

(C_8_H_15_N_2_)[Ni(C_4_N_2_S_2_)_2_]
*M* *_r_* = 478.31
Monoclinic, 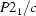

*a* = 10.650 (2) Å
*b* = 7.3924 (13) Å
*c* = 26.691 (5) Å
β = 93.463 (5)°
*V* = 2097.5 (7) Å^3^
*Z* = 4
Mo *K*α radiation
μ = 1.34 mm^−1^
*T* = 298 K
0.40 × 0.20 × 0.15 mm

#### Data collection

Bruker SMART CCD area-detector diffractometer
Absorption correction: multi-scan (*SADABS*; Bruker, 2000 ▶) *T*_min_ = 0.733, *T*_max_ = 0.818
19183 measured reflections
3824 independent reflections
3246 reflections with *I* > 2σ(*I*)
*R*_int_ = 0.044

#### Refinement

*R*[*F* ^2^ > 2σ(*F* ^2^)] = 0.050
*wR*(*F* ^2^) = 0.099
*S* = 1.16
3824 reflections
247 parameters
H-atom parameters constrained
Δρ_max_ = 0.35 e Å^−3^
Δρ_min_ = −0.34 e Å^−3^

### 

Data collection: *SMART* (Bruker, 2000 ▶); cell refinement: *SAINT* (Bruker, 2000 ▶); data reduction: *SAINT*; program(s) used to solve structure: *SHELXTL* (Sheldrick, 2008 ▶); program(s) used to refine structure: *SHELXTL*; molecular graphics: *SHELXTL*; software used to prepare material for publication: *SHELXTL*.

## Supplementary Material

Crystal structure: contains datablock(s) global, I. DOI: 10.1107/S1600536811046824/is2799sup1.cif

Structure factors: contains datablock(s) I. DOI: 10.1107/S1600536811046824/is2799Isup2.hkl

Additional supplementary materials:  crystallographic information; 3D view; checkCIF report

## Figures and Tables

**Table 1:** Hydrogen-bond geometry (Å, °)

*D*—H⋯*A*	*D*—H	H⋯*A*	*D*⋯*A*	*D*—H⋯*A*
C13—H13⋯N4^i^	0.93	2.57	3.446 (5)	158
C15—H15*B*⋯N2^ii^	0.96	2.58	3.443 (5)	149

Symmetry codes: (i) 
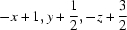
; (ii) 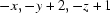.

**Fig. 1.**
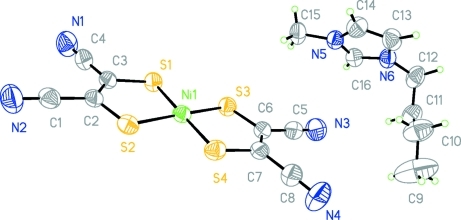
The molecular structure of (I), showing the atom-numbering scheme and displacement ellipsoids at the 30% probability level.

## supplementary crystallographic information

### Comment

Bis(1,2-dithiolene) complexes of transition metals have been widely studied due to their novel properties and applications in the areas of near-infrared (near-IR) dyes, conducting, magnetic and nonlinear optical materials (Nishijo *et al.*, 2000; Ni *et al.*, 2005). The behavior of the packing structure for bis(1,2-dithiolene) complexes monoanions was strongly affected by the type of counterions. Herein we introduce a flexible organic cation into dithiolene monoanions system and report the crystal structure of the title compound (I).

The molecular structure of (I) is illustrated in Fig. 1. The asymmmetric unit comprises one [Ni(mnt)_2_]^-^ monoanion and one 1-methyl-3-butyl-imidazolinium cation. The Ni ion in the [Ni(mnt)_2_]^-^ anion is coordinated by four S atoms of two mnt^2-^ ligands, and exhibits square-planar coordination geometry, and their molecular planes defined by four coordination S atom are approximately parallel to each other. The bond lengths and angles of anions are in good agreement with the various [Ni(mnt)_2_]^-^ compounds (Ni *et al.*, 2004; Ren *et al.*, 2004, 2008; Duan *et al.*, 2010). The cation adopts a bent conformation, its hydrocarbon chain slightly disrupted close to the imidazole ring with an almost completely *trans*-planar conformation.

### Experimental

Disodium maleonitriledithiolate (1.5 mmol) and nickel chloride hexahydrate (0.8 mmol) were mixed under stirring in water (20 mL) at room temperature. Subsequently, a solution of 1-methyl-3-butyl-imidazolinium bromide (1.5 mmol) in water (10 mL) was added to the mixture, and the red precipitate that was immediately formed was filtered off, and washed with water. Then, a methanol solution of I_2_ (0.8 mmol) was slowly added to a red precipitate, after stirred for 20 min, the mixture was allowed standing overnight. The microcryatalline formed and the crude product was recrystallized in acetone to give black block crystals.

### Refinement

H atoms were placed in geometrically idealized positions with 0.97 Å for methylene H atoms and 0.96 Å for methyl H atoms, respectively, and were refined as riding atoms with *U*_iso_(H) = 1.2*U*_eq_(C) for methylene H atoms and *U*_iso_(H) = 1.5*U*_eq_(C) for methyl H atoms.

### Crystal data

**Table:**

(C_8_H_15_N_2_)[Ni(C_4_N_2_S_2_)_2_]	*F*(000) = 980.0
*M**_r_* = 478.31	*D*_x_ = 1.515 Mg m^−^^3^
Monoclinic, *P*2_1_/*c*	Mo *K*α radiation, λ = 0.71070 Å
Hall symbol: -p 2Ybc	Cell parameters from 778 reflections
*a* = 10.650 (2) Å	θ = 2.6–21.2°
*b* = 7.3924 (13) Å	µ = 1.34 mm^−^^1^
*c* = 26.691 (5) Å	*T* = 298 K
β = 93.463 (5)°	Block, black
*V* = 2097.5 (7) Å^3^	0.40 × 0.20 × 0.15 mm
*Z* = 4	

### Data collection

**Table:**

Bruker SMART CCD area-detector diffractometer	3824 independent reflections
Radiation source: fine-focus sealed tube	3246 reflections with *I* > 2σ(*I*)
graphite	*R*_int_ = 0.044
φ and ω scans	θ_max_ = 25.4°, θ_min_ = 3.1°
Absorption correction: multi-scan (*SADABS*; Bruker, 2000)	*h* = −12→12
*T*_min_ = 0.733, *T*_max_ = 0.818	*k* = −8→7
19183 measured reflections	*l* = −32→30

### Refinement

**Table:**

Refinement on *F*^2^	Primary atom site location: structure-invariant direct methods
Least-squares matrix: full	Secondary atom site location: difference Fourier map
*R*[*F*^2^ > 2σ(*F*^2^)] = 0.050	Hydrogen site location: inferred from neighbouring sites
*wR*(*F*^2^) = 0.099	H-atom parameters constrained
*S* = 1.16	*w* = 1/[σ^2^(*F*_o_^2^) + (0.0319*P*)^2^ + 1.4061*P*] where *P* = (*F*_o_^2^ + 2*F*_c_^2^)/3
3824 reflections	(Δ/σ)_max_ = 0.001
247 parameters	Δρ_max_ = 0.35 e Å^−^^3^
0 restraints	Δρ_min_ = −0.34 e Å^−^^3^

### Special details

**Table:**

Geometry. All e.s.d.'s (except the e.s.d. in the dihedral angle between two l.s. planes) are estimated using the full covariance matrix. The cell e.s.d.'s are taken into account individually in the estimation of e.s.d.'s in distances, angles and torsion angles; correlations between e.s.d.'s in cell parameters are only used when they are defined by crystal symmetry. An approximate (isotropic) treatment of cell e.s.d.'s is used for estimating e.s.d.'s involving l.s. planes.
Refinement. Refinement of *F*^2^ against ALL reflections. The weighted *R*-factor *wR* and goodness of fit *S* are based on *F*^2^, conventional *R*-factors *R* are based on *F*, with *F* set to zero for negative *F*^2^. The threshold expression of *F*^2^ > σ(*F*^2^) is used only for calculating *R*-factors(gt) *etc*. and is not relevant to the choice of reflections for refinement. *R*-factors based on *F*^2^ are statistically about twice as large as those based on *F*, and *R*- factors based on ALL data will be even larger.

### Fractional atomic coordinates and isotropic or equivalent isotropic displacement parameters (Å^2^)

**Table:**

	*x*	*y*	*z*	*U*_iso_*/*U*_eq_	
Ni1	0.09847 (4)	0.70716 (6)	0.503296 (15)	0.04030 (15)	
S1	0.13066 (8)	0.76184 (13)	0.42646 (3)	0.0483 (2)	
S2	−0.09351 (8)	0.79241 (12)	0.49400 (3)	0.0456 (2)	
S3	0.28875 (9)	0.61212 (14)	0.51160 (3)	0.0507 (3)	
S4	0.06746 (9)	0.65718 (13)	0.58064 (3)	0.0486 (2)	
N1	−0.0210 (4)	0.9489 (6)	0.31048 (14)	0.0832 (12)	
N2	−0.3277 (3)	0.9738 (5)	0.40060 (15)	0.0789 (11)	
N3	0.5208 (4)	0.4175 (6)	0.60350 (16)	0.0882 (13)	
N4	0.2194 (4)	0.4677 (6)	0.69608 (14)	0.0956 (14)	
N5	0.6606 (3)	0.8606 (4)	0.59871 (10)	0.0476 (7)	
N6	0.7729 (3)	0.7519 (4)	0.66100 (11)	0.0510 (8)	
C1	−0.2310 (4)	0.9224 (5)	0.41389 (15)	0.0532 (10)	
C2	−0.1106 (3)	0.8554 (4)	0.43214 (13)	0.0425 (8)	
C3	−0.0107 (3)	0.8444 (4)	0.40249 (13)	0.0432 (8)	
C4	−0.0187 (3)	0.9029 (6)	0.35110 (15)	0.0550 (10)	
C5	0.4244 (4)	0.4750 (6)	0.59122 (15)	0.0596 (11)	
C6	0.3061 (3)	0.5491 (5)	0.57365 (13)	0.0477 (9)	
C7	0.2078 (3)	0.5671 (5)	0.60397 (13)	0.0478 (9)	
C8	0.2156 (4)	0.5117 (6)	0.65538 (16)	0.0612 (11)	
C9	0.5952 (6)	0.3532 (9)	0.7533 (3)	0.157 (3)	
H9A	0.5596	0.2909	0.7243	0.236*	
H9B	0.5289	0.3953	0.7732	0.236*	
H9C	0.6487	0.2722	0.7729	0.236*	
C10	0.6682 (5)	0.5058 (7)	0.7376 (2)	0.0941 (17)	
H10A	0.7005	0.5701	0.7673	0.113*	
H10B	0.6124	0.5875	0.7185	0.113*	
C11	0.7771 (4)	0.4598 (6)	0.70632 (16)	0.0681 (12)	
H11A	0.8329	0.3767	0.7249	0.082*	
H11B	0.7455	0.3992	0.6759	0.082*	
C12	0.8509 (4)	0.6250 (6)	0.69236 (15)	0.0665 (12)	
H12A	0.8823	0.6863	0.7227	0.080*	
H12B	0.9226	0.5876	0.6742	0.080*	
C13	0.7196 (4)	0.9081 (6)	0.67674 (14)	0.0592 (10)	
H13	0.7301	0.9583	0.7087	0.071*	
C14	0.6497 (4)	0.9761 (5)	0.63792 (14)	0.0573 (10)	
H14	0.6027	1.0821	0.6377	0.069*	
C15	0.6000 (4)	0.8833 (6)	0.54840 (13)	0.0624 (11)	
H15A	0.6348	0.9871	0.5326	0.094*	
H15B	0.5112	0.9002	0.5509	0.094*	
H15C	0.6144	0.7774	0.5287	0.094*	
C16	0.7359 (3)	0.7259 (5)	0.61352 (13)	0.0493 (9)	
H16	0.7588	0.6291	0.5937	0.059*	

### Atomic displacement parameters (Å^2^)

**Table:**

	*U*^11^	*U*^22^	*U*^33^	*U*^12^	*U*^13^	*U*^23^
Ni1	0.0425 (3)	0.0390 (3)	0.0395 (3)	−0.0044 (2)	0.0042 (2)	−0.00279 (19)
S1	0.0432 (5)	0.0599 (6)	0.0422 (5)	0.0011 (4)	0.0067 (4)	0.0003 (4)
S2	0.0430 (5)	0.0454 (5)	0.0490 (5)	−0.0041 (4)	0.0084 (4)	−0.0038 (4)
S3	0.0461 (5)	0.0585 (6)	0.0476 (5)	−0.0001 (5)	0.0036 (4)	0.0011 (5)
S4	0.0529 (6)	0.0490 (6)	0.0446 (5)	−0.0023 (4)	0.0076 (4)	0.0001 (4)
N1	0.076 (3)	0.118 (3)	0.054 (2)	−0.009 (2)	−0.005 (2)	0.012 (2)
N2	0.052 (2)	0.081 (3)	0.102 (3)	0.006 (2)	−0.004 (2)	−0.003 (2)
N3	0.056 (2)	0.100 (3)	0.107 (3)	−0.001 (2)	−0.013 (2)	0.027 (3)
N4	0.121 (4)	0.112 (4)	0.053 (2)	−0.006 (3)	−0.001 (2)	0.019 (2)
N5	0.0408 (17)	0.058 (2)	0.0440 (17)	−0.0021 (15)	0.0029 (14)	0.0024 (15)
N6	0.0439 (18)	0.064 (2)	0.0444 (18)	−0.0002 (16)	−0.0010 (14)	0.0042 (16)
C1	0.051 (2)	0.047 (2)	0.061 (2)	−0.0069 (19)	0.002 (2)	−0.0057 (19)
C2	0.042 (2)	0.0330 (18)	0.052 (2)	−0.0043 (15)	−0.0030 (17)	−0.0037 (16)
C3	0.044 (2)	0.040 (2)	0.045 (2)	−0.0047 (16)	−0.0017 (16)	−0.0040 (16)
C4	0.048 (2)	0.065 (3)	0.051 (2)	−0.0016 (19)	−0.0032 (19)	−0.003 (2)
C5	0.054 (3)	0.060 (3)	0.064 (3)	−0.007 (2)	−0.002 (2)	0.011 (2)
C6	0.051 (2)	0.044 (2)	0.048 (2)	−0.0071 (17)	−0.0043 (18)	0.0020 (17)
C7	0.056 (2)	0.045 (2)	0.041 (2)	−0.0131 (17)	−0.0027 (18)	0.0002 (17)
C8	0.069 (3)	0.061 (3)	0.052 (3)	−0.007 (2)	−0.003 (2)	0.004 (2)
C9	0.149 (6)	0.121 (6)	0.211 (8)	0.013 (5)	0.089 (6)	0.062 (5)
C10	0.081 (3)	0.093 (4)	0.112 (4)	0.006 (3)	0.033 (3)	0.029 (3)
C11	0.070 (3)	0.072 (3)	0.062 (3)	0.016 (2)	0.003 (2)	0.012 (2)
C12	0.048 (2)	0.091 (3)	0.060 (2)	0.009 (2)	−0.001 (2)	0.019 (2)
C13	0.066 (3)	0.065 (3)	0.047 (2)	−0.003 (2)	0.004 (2)	−0.009 (2)
C14	0.060 (3)	0.055 (2)	0.057 (2)	0.007 (2)	0.007 (2)	0.001 (2)
C15	0.050 (2)	0.086 (3)	0.050 (2)	0.000 (2)	−0.0053 (19)	0.005 (2)
C16	0.044 (2)	0.057 (2)	0.047 (2)	0.0003 (18)	0.0053 (17)	−0.0027 (18)

### Geometric parameters (Å, °)

**Table:**

Ni1—S1	2.1382 (10)	C6—C7	1.368 (5)
Ni1—S2	2.1395 (10)	C7—C8	1.429 (5)
Ni1—S4	2.1424 (10)	C9—C10	1.446 (7)
Ni1—S3	2.1436 (11)	C9—H9A	0.9600
S1—C3	1.712 (4)	C9—H9B	0.9600
S2—C2	1.715 (4)	C9—H9C	0.9600
S3—C6	1.719 (4)	C10—C11	1.509 (6)
S4—C7	1.718 (4)	C10—H10A	0.9700
N1—C4	1.135 (5)	C10—H10B	0.9700
N2—C1	1.134 (5)	C11—C12	1.510 (6)
N3—C5	1.140 (5)	C11—H11A	0.9700
N4—C8	1.133 (5)	C11—H11B	0.9700
N5—C16	1.324 (4)	C12—H12A	0.9700
N5—C14	1.361 (5)	C12—H12B	0.9700
N5—C15	1.464 (4)	C13—C14	1.337 (5)
N6—C16	1.318 (4)	C13—H13	0.9300
N6—C13	1.364 (5)	C14—H14	0.9300
N6—C12	1.479 (5)	C15—H15A	0.9600
C1—C2	1.433 (5)	C15—H15B	0.9600
C2—C3	1.366 (5)	C15—H15C	0.9600
C3—C4	1.436 (5)	C16—H16	0.9300
C5—C6	1.428 (5)		
			
S1—Ni1—S2	92.32 (4)	H9A—C9—H9C	109.5
S1—Ni1—S4	178.96 (4)	H9B—C9—H9C	109.5
S2—Ni1—S4	87.75 (4)	C9—C10—C11	115.5 (5)
S1—Ni1—S3	87.47 (4)	C9—C10—H10A	108.4
S2—Ni1—S3	177.89 (4)	C11—C10—H10A	108.4
S4—Ni1—S3	92.50 (4)	C9—C10—H10B	108.4
C3—S1—Ni1	103.73 (12)	C11—C10—H10B	108.4
C2—S2—Ni1	103.70 (12)	H10A—C10—H10B	107.5
C6—S3—Ni1	103.54 (13)	C12—C11—C10	112.5 (4)
C7—S4—Ni1	103.58 (12)	C12—C11—H11A	109.1
C16—N5—C14	108.7 (3)	C10—C11—H11A	109.1
C16—N5—C15	125.8 (3)	C12—C11—H11B	109.1
C14—N5—C15	125.5 (3)	C10—C11—H11B	109.1
C16—N6—C13	108.3 (3)	H11A—C11—H11B	107.8
C16—N6—C12	125.2 (3)	N6—C12—C11	111.7 (3)
C13—N6—C12	126.3 (3)	N6—C12—H12A	109.3
N2—C1—C2	178.1 (5)	C11—C12—H12A	109.3
C3—C2—C1	122.4 (3)	N6—C12—H12B	109.3
C3—C2—S2	120.0 (3)	C11—C12—H12B	109.3
C1—C2—S2	117.5 (3)	H12A—C12—H12B	107.9
C2—C3—C4	122.1 (3)	C14—C13—N6	107.6 (3)
C2—C3—S1	120.2 (3)	C14—C13—H13	126.2
C4—C3—S1	117.7 (3)	N6—C13—H13	126.2
N1—C4—C3	177.8 (4)	C13—C14—N5	106.9 (4)
N3—C5—C6	177.3 (5)	C13—C14—H14	126.5
C7—C6—C5	122.3 (3)	N5—C14—H14	126.5
C7—C6—S3	120.1 (3)	N5—C15—H15A	109.5
C5—C6—S3	117.5 (3)	N5—C15—H15B	109.5
C6—C7—C8	122.5 (4)	H15A—C15—H15B	109.5
C6—C7—S4	120.2 (3)	N5—C15—H15C	109.5
C8—C7—S4	117.3 (3)	H15A—C15—H15C	109.5
N4—C8—C7	178.7 (5)	H15B—C15—H15C	109.5
C10—C9—H9A	109.5	N6—C16—N5	108.5 (3)
C10—C9—H9B	109.5	N6—C16—H16	125.7
H9A—C9—H9B	109.5	N5—C16—H16	125.7
C10—C9—H9C	109.5		
			
S3—Ni1—S1—C3	179.03 (12)	C5—C6—C7—S4	−179.9 (3)
S1—Ni1—S2—C2	−0.41 (12)	S3—C6—C7—S4	−1.4 (4)
S4—Ni1—S2—C2	178.55 (12)	Ni1—S4—C7—C6	1.9 (3)
S1—Ni1—S3—C6	179.74 (13)	Ni1—S4—C7—C8	−177.6 (3)
S4—Ni1—S3—C6	0.78 (13)	C9—C10—C11—C12	−178.6 (5)
S2—Ni1—S4—C7	176.57 (13)	C16—N6—C12—C11	−72.9 (5)
Ni1—S2—C2—C3	−0.7 (3)	C13—N6—C12—C11	102.1 (5)
Ni1—S2—C2—C1	−179.3 (2)	C10—C11—C12—N6	−62.5 (5)
C1—C2—C3—C4	1.3 (5)	C16—N6—C13—C14	0.0 (4)
S2—C2—C3—C4	−177.2 (3)	C12—N6—C13—C14	−175.7 (3)
C1—C2—C3—S1	−179.6 (3)	N6—C13—C14—N5	0.1 (4)
S2—C2—C3—S1	1.8 (4)	C16—N5—C14—C13	−0.2 (4)
Ni1—S1—C3—C2	−1.9 (3)	C15—N5—C14—C13	−179.5 (3)
Ni1—S1—C3—C4	177.2 (3)	C13—N6—C16—N5	−0.1 (4)
Ni1—S3—C6—C7	0.2 (3)	C12—N6—C16—N5	175.6 (3)
Ni1—S3—C6—C5	178.7 (3)	C14—N5—C16—N6	0.2 (4)
C5—C6—C7—C8	−0.5 (6)	C15—N5—C16—N6	179.6 (3)
S3—C6—C7—C8	178.0 (3)		

### Hydrogen-bond geometry (Å, °)

**Table:**

*D*—H···*A*	*D*—H	H···*A*	*D*···*A*	*D*—H···*A*
C13—H13···N4^i^	0.93	2.57	3.446 (5)	158
C15—H15B···N2^ii^	0.96	2.58	3.443 (5)	149

Symmetry codes: (i) −*x*+1, *y*+1/2, −*z*+3/2; (ii) −*x*, −*y*+2, −*z*+1.
